# Grade 1 Endometrioid Endometrial Carcinoma Presenting with Pelvic Bone Metastasis: A Case Report and Review of the Literature

**DOI:** 10.1155/2013/807205

**Published:** 2013-04-22

**Authors:** My-Linh T. Nguyen, Christopher J. LaFargue, Tarah L. Pua, Sean S. Tedjarati

**Affiliations:** Department of Obstetrics and Gynecology, Westchester Medical Center of New York Medical College, Munger Pavilion, Room 617, Valhalla, NY 10595, USA

## Abstract

Most grade 1 endometrioid endometrial cancers are confined to the uterus at the time of diagnosis and confer a good prognosis. Rarely will a grade 1 endometrioid endometrial carcinoma present with distant metastasis, especially to the bone. We present the case of a 56-year-old woman with postmenopausal bleeding and right hip pain due to metastatic grade 1 endometrioid uterine cancer invading into the right ischium. We discuss treatment options as well as provide a review of prior published reports on bony metastasis in grade 1 endometrioid endometrial cancers. To date, this case is one of 10 others which demonstrates that even a well-differentiated, low-grade endometrioid endometrial carcinoma can progress in a highly aggressive manner.

## 1. Introduction

Endometrial cancer is the most common malignancy of the female genital tract and the fourth most common cancer in women [1]. Factors which typically confer a better prognosis and outcome are surgical stage I, low histologic grade, nonserous or nonclear cell morphology, and superficial or no invasion of the myometrium [1]. While surgical staging remains the primary modality for determining the extent of disease, the histological grade is an important prognostic indicator and is highly predictive in determining propensity for metastasis [1]. In the largest series to date on grade 1 endometrioid endometrial cancers, the incidence of pelvic lymph node involvement, pelvic metastasis, and distant metastasis specific to grade 1 tumors is estimated at 3.3%, 4.6%, and 2.4%, respectively [2]. Considering the relative rarity of distant metastasis in grade 1 endometrial carcinomas, we present a case of grade 1 endometrioid endometrial cancer presenting with bone metastasis to the ischium.

## 2. Case

**Figure 1:**
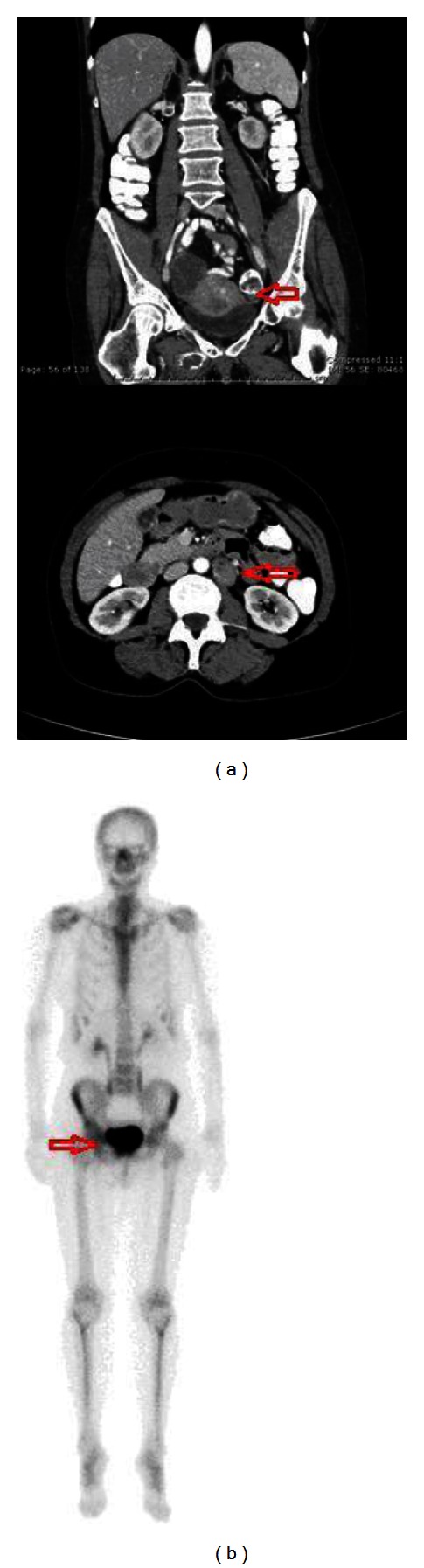
MRI (a) and bone scan (b) of uterine mass metastatic to bone.

A 56 year-old para 2002 presented with a 30-pound weight loss, postmenopausal bleeding, and right-sided hip pain for two years and increasing difficulty walking. Upon exam, a tender right pubic bone, a 4 cm palpable nodule at the anterior vaginal wall, and a 2 cm nodule at the cervicovaginal junction were noted. These nodules and the endometrium were biopsied. Pathology revealed FIGO grade 1 endometrioid endometrial adenocarcinoma (EEC) from all biopsy sites. Immunohistochemical staining revealed tumor cells positive for estrogen receptor (ER) and progesterone receptor (PR) and scatteredly positive for p53. Ki-67 showed high proliferative index. PET/CT imaging demonstrated enlarged retroperitoneal lymph nodes along the aorta and inferior vena cava (SUV > 8). The uterus (SUV > 16) contained a soft tissue lesion invading the right inferior pubic ramus (SUV > 15). Pelvic MRI revealed a thickened endometrium with complex enhancement extending into the lower uterine segment and to the serosal surface (Figure 1(a)). Lesions suspicious for metastasis were noted to infiltrate into the adjacent adductor musculature. A bone scan revealed increased radiotracer uptake within the right ischium extending into the superior pubic ramus and the right pubic bone (Figure 1(b)). A CAT scan of the chest was unremarkable for metastasis.

A soft tissue core biopsy of the right pelvic region revealed adenocarcinoma consistent with a primary endometrial tumor. The patient was diagnosed with stage IVB uterine cancer, and she subsequently underwent palliative radiation, chemotherapy with IV cisplatin and zoledronic acid. Three months following initiation of therapy, a CT of the chest, abdomen, and pelvis showed newly enlarged right common iliac lymph nodes and left portacaval and left periaortic lymph nodes that had increased in size and number. The pubic bone contained an expansile destructive lesion measuring 7 cm, completely replacing the right inferior pubic ramus and invading the right adductor musculature. Despite her advancing disease, the patient had symptomatic improvement of her pelvic pain.

After completing radiation and six cycles of IV cisplatin, the patient is currently undergoing a planned additional six cycles of IV carboplatin and paclitaxel. She is alive at 9 months following her initial diagnosis with progression of disease.

## 3. Discussion

Metastatic endometrial cancer lesions are predominantly found in the lymph nodes, omentum, lungs, and liver. The spread is typically from direct invasion or via the lymphovascular pathway [3]. Endometrial cancer with metastasis to bone has been reported to occur in 2–6% of all metastatic endometrial cancers [3]. Of the reported cases of bony metastases, the most common locations have involved the appendicular skeleton with a high surgical stage and grade. Although hematogenous dissemination is the most common route of bony metastasis, we suspect that the patient's tumor in this case invaded by direct extension. Although likely underreported, to the best of our knowledge, only 10 other cases of grade I endometrial cancer with bony metastasis have been reported (Table 1). The most common location of metastases was in the axial skeleton (vertebrae and pelvis). There is no consensus on the standard treatment of stage IVB endometrioid endometrial carcinoma. Prognosis is poor and the treatment is predominantly palliative. A review of the literature reveals that the most common treatment for metastases to the bone involves surgical removal of the lesion (if possible), site-directed radiation therapy, and IV chemotherapy [4]. Considering that surgical resection of the bony metastasis was not an option for the patient in this case, our plan was to proceed with pelvic radiation and chemotherapy. In addition, bisphosphonates were added as they have been shown to have a modest improvement in skeletal-related event-free survival in one report [4]. 

## 4. Conclusion

Although most cases of grade 1 endometrioid endometrial carcinoma do not behave aggressively, this case demonstrates the potential for progression of grade 1 disease. It is evident that, in rare instances, even low-grade, well-differentiated endometrial adenocarcinomas can progress in a highly aggressive manner.

## Figures and Tables

**Table 1:** Bone metastasis in patients with grade 1 endometrial cancer.

Patient	Author	Year	Age	Initial stage	Initial treatment	Months diagnosis to treatment	Site of metastasis	Treatment of metastasis	Followup (months)	Dead
1	Nishida et al. [5]	1994	61	IIIB	N/A		Calcaneus L	N/A	N/A	N/A
2	Petru et al. [6]	1995	61	IVB	Lower leg amputation, TAH/BSO/PPLND	0	Tarsus L	SX/QMT/MG	10	No
3	Arnold et al. [7]	2003	63	IVB	TAH/BSO	0	Thoracic vert	RDT/MG	60	No
4	Uharček et al. [8]	2006	67	IVB	TAH/BSO/PPLND/lower leg amputation, QMT, progesterone acetate	0	Calcaneus, talus, metatarsus		20	Yes
5	Giannakopoulos et al. [9]	2006	68	IVA	N/A	0	R ischium	RDT	36	Yes
6	Kaya et al. [10]	2007	70	IVA	Endo CA not diagnosed until after 1 year of mets	?	Tibia	RDT	47	Yes
7	Albareda et al. [3]	2008	62	IA	TAH/BSO/staging	37	Sacrum	SX/RDT/MG	16	No
8	Kehoe et al. [11]	2010	61	IIIA	TAH/BSO/WPRT	44	Vert	RDT/SX	12	Yes
9	Kehoe et al. [11]	2010	65	IIIB	WPRT/IVRT	7	Tibia, femur	RDT/SX	42	Yes
10	Kehoe et al. [11]	2010	55	Unstaged	TAH/BSO/staging	25	Pelvis, sacrum, vertebrae, rib	QMT	7	Yes
11	Present case	2012	56	IVB	N/A	0	R pubic ramus and ischium	RDT/QMT/BP	9	No

TAH: total abdominal hysterectomy, BSO: bilateral salpingoophorectomy, PPLND: pelvic and paraaortic lymph node dissection, SX: surgery, QMT: chemotherapy, RDT: radiation therapy, BP: bisphosphonates, MG: medroxyprogesterone acetate.

L: left and R: right.

## References

[1] Rose PG (1996). Endometrial carcinoma. *The New England Journal of Medicine*.

[2] Chan JK, Kapp DS, Cheung MK (2008). Prognostic factors and risk of extrauterine metastases in 3867 women with grade 1 endometrioid corpus cancer. *American Journal of Obstetrics and Gynecology*.

[3] Albareda J, Herrera M, Lopez Salva A, Garcia Donas J, Gonzalez R (2008). Sacral metastasis in a patient with endometrial cancer: case report and review of the literature. *Gynecologic Oncology*.

[4] Shigemitsu A, Furukawa N, Koike N, Kobayashi H (2010). Endometrial cancer diagnosed by the presence of bone metastasis and treated with zoledronic acid: a case report and review of the literature. *Case Reports in Oncology*.

[5] Nishida Y, Hayata T, Miyakawa I (1994). Metastatic calcaneal adenocarcinoma in a patient with uterine carcinoma. *International Journal of Gynecology and Obstetrics*.

[6] Petru E, Malleier M, Lax S (1995). Solitary metastasis in the tarsus preceding the diagnosis of primary endometrial cancer—a case report. *European Journal of Gynaecological Oncology*.

[7] Arnold J, Charters D, Perrin L (2003). Prolonged survival time following initial presentation with bony metastasis in stage IVb endometrial carcinoma. *Australian and New Zealand Journal of Obstetrics and Gynaecology*.

[8] Uharček P, Mlynček M, Ravinger J (2006). Endometrial adenocarcinoma presenting with an osseous metastasis. *Gynecologic and Obstetric Investigation*.

[9] Giannakopoulos CK, Kyriakidou GK, Toufexi GE (2006). Bone metastasis as a presenting feature of endometrial adenocarcinoma: case report and literature review. *European Journal of Gynaecological Oncology*.

[10] Kaya A, Olmezoglu A, Eren CS (2007). Solitary bone metastasis in the tibia as a presenting sign of endometrial adenocarcinoma: a case report and the review of the literature. *Clinical and Experimental Metastasis*.

[11] Kehoe SM, Zivanovic O, Ferguson SE, Barakat RR, Soslow RA (2010). Clinicopathologic features of bone metastases and outcomes in patients with primary endometrial cancer. *Gynecologic Oncology*.

